# Covid-19 and female migrants: policy challenges and multiple vulnerabilities

**DOI:** 10.1186/s40878-022-00295-z

**Published:** 2022-06-01

**Authors:** Dudziro Nhengu

**Affiliations:** 311 Stonebridge Road, Parktown, Harare, Zimbabwe

**Keywords:** Covid-19, Gender, Women and girls, Migrants

## Abstract

To what extent has Covid-19 policy responses exacerbated the already existing multiple vulnerabilities of female migrants in Southern Africa? Using strategic conversations, the paper explores personal experiences of key conversants, to explore how gender blind policy responses to the pandemic have heightened female migrants’ socio-economic challenges. The paper recommends gender sensitive and context specific policy responses to mitigate the existing socio-economic challenges.

## Introduction

To what extent have Covid-19 policy responses exacerbated the already existing multiple vulnerabilities of female migrants in Southern Africa? This paper examines the degree to which Covid-19 policy responses have exacerbated pre-existing multiple vulnerabilities of female Zimbabwean migrants working in Botswana and South Africa. Cross-regional migration prototypes have been a common feature in Southern Africa since the nineteenth century, owing to the employment prospects in mines, commercial farms and plantations (Crush et al., 2005; Oucho, 2005). A current shift in these migration patterns which exudes a progressive intensification of mobility in the region, a trend determined by among other factors, increased opportunities for legalised cross-border trading regionally and globally, increasing levels of unemployment and poverty in home countries, forced migration and displacements emanating from civil wars, political conflicts, climate change and related environmental conflicts has been noted (Crush et al., 2004). In Zimbabwe, the period from the early 90 s witnessed an upsurge in the numbers of people, especially women, leaving their homes both as documented and undocumented migrants, to settle either for employment or for refuge or for both, mostly in South Africa and Botswana, the two countries that are considered as having a middle-class income status, stable democratic institutions and a relatively industrialized economy (Lekalake, 2014; Muller, 2021). These movements are the women’s attempts to address their spatial and social marginalisation in an economically and politically challenged society, through seeking out better jobs and new settlements either as professionals and students or as general workers—including domestic workers, factory cleaners and home based care takers (Suhardiman et al., 2021).

Demographically women are in the majority of migrants with low educational and skills levels, who relocate illegally, driven mostly by economic necessity, factors of which inhibit them from acquiring required documentation. Lack of information on how to get registered for formal migration likewise confers an illegal status on them. As a result, the majority of women militate towards dirty, dangerous and difficult/demeaning jobs (3D jobs)like care and domestic work, where there is an absence of effective safeguards to protect their rights. According to the Maison Law Professional Legal Corporation (https://maisonlaw.com/2018/12/the-unsafe-3-d-jobs-of-migrant-workers/), a 3D job is one that is dirty, dangerous and demeaning, and cane range from picking fruit in an apple orchard to scrubbing dirty floors. As such female migrants in this category of 3D jobs further face exploitative practices from recruitment agencies and employers who don’t give them good enough working conditions to enable the full realization of their right to health (Grover, 2013). Despite their migrant status, research has ample evidence to prove that relocating to foreign countries brought marked improvements in the female migrants’ personal incomes, health and education prospects in the diaspora—including better life chances like improved monthly incomes, better food security, better education and personal home ownership for their families back home, what Sakdapolrak (2014) and Vicol (2019) refer to as ‘livelihood trajectories’.

On one hand there is ample empirical evidence to prove that migration can empower migrants, enhance human capital, raise incomes, develop both sending and receiving communities, lift people and places out of poverty, and engender ‘good change’ world over (UNDP, 2009). On the other hand however, the social challenges like disruptions in family life; including the insecurities wrought by child headed households in the event where single mothers leave their families in search of greener pastures, the disruptions in marriage life when spouses separate for abnormally long periods of time and the difficulties that the migrants face in transit as well as in trying to adapt to the new environment in their countries of destination (Derks, 2013). When they reach the host countries, migrant workers endure setbacks such as long physical and verbal abuse, conditional ties that limit their potential to a particular employer, restrictions in movement and wages that are perceived as unfair when weighed against the work undertaken (Bélanger, 2014). For Jain and Sharma (2018) and Sharma (2011), migration can be described as disempowering, deskilling and immiserising, and occasionally a driver of negative change.

While migrating to a foreign country for work may pose risks and vulnerabilities to many migrant workers of all sexes in general,^1^ the situation of female migrants is doubly worse than that of their male counterparts. As such, the concept of feminised vulnerabilities unlocks key aspects of contemporary migration discourse which in turn have a link to the health care specific need of women in times of Covid-19, and the major issues center around women’s sexual and reproductive health rights during transit and beyond (Grotti et al. 2019). Owing to their biological make-up in a largely hetero-normative context, women are more prone to sexual and gender based violations, including rape, during transit and beyond. Such violations have domino effects on women’s health, status and well-being such as sexually transmitted infections, unwanted pregnancies and inadequate health care and legal services. From a gender perspective, female migrant vulnerabilities are a form of structural violence and gender discrimination perpetrated on human beings by policy makers both in their states of origin and on the global health front, and can be better understood within the ambit of the theory of social determinants of health (SDH) (Gumà et al., 2019). Some SDH theories focus on the ‘social production of disease’ and the need to address the structural causes of inequality as a prerogative in addressing health vulnerabilities, hence the need to focus on gender sensitive policy reform. SDH theories also explore the effects of poverty and inequality on individuals as the causes of harmful exposures and psychosocial stress that, in turn, cause greater susceptibility to poor health (Gumà et al. 2019). Women are susceptible to various forms of stresses and mental disorders than men, owing to a number of reasons.

Furthermore, simply being in the majority of migrant populations who occupy insecure, hazardous and poorly regulated 3D jobs in the absence of effective safeguards to protect their rights further exposes female migrants to more exploitative practices as recruitment agencies and employers may fail to provide human-rights friendly conditions to enable them the full realization of their right to work, health and socio-economic well-being (Grover, 2013). These feminised vulnerabilities of female migrants have been made worse by the advent of Covid-19. Ironically, the pandemic surfaced at a time when the globe was already reeling from the negative consequences of gender blind migration policies which promote inequalities in health provision circles, fuel negative health outcomes for development planners, while increasing health risks for migrant populations (Nhengu, 2021). Existing gender blind institutional frameworks likewise influenced the nature of Covid-19 policies that development planners put in place, further compounding the vulnerabilities of female migrants.

Ambivalences characterise the narrative of Covid-19 and development globally. On one hand, the World Bank provides evidence that Covid-19 has not brought a push back to the narrative of migration and development perse, as in general, global financial remittances even increased during the pandemic (World Bank, 2021). In Sub-Sahara Africa, remittances increased by 2.3 percent, a sure sign of economic resilience. On the other hand however, a gender and intersectionality analysis requires a deepened and practical understanding of how increases in remittances alone may not necessarily translate to matched gains and improved livelihoods for undocumented women migrants both vertically and horizontally, and in some instances for documented migrants too, depending on their geographies of insecurity. In countries like Zimbabwe for example, the argument that the formal record of remittances increased during the lockdown period may be justified by the fact that other informal means of sending money down from South Africa and Botswana to Zimbabwe had shut down, following for example, the closure of cross-border bus services. Likewise, most migrant workers found themselves locked down in foreign countries, unable to make monthly trips back home to bring the remittances in person as usual. This meant that where migrant workers could send money informally through the bus services, the lockdown required them to formally send money by Western Union or Money Gram, thus tilting the scale of formal remittances. The sum total of these arguments is that gender sensitive migration policy and practice in the face of Covid-19 requires an increasing recognition of, and a desire to understand the multifaceted and complex relationship between migration, covid 19 policy and economic development, but more so to understand the various variables that intersect to render female migrant workers in Africa at different levels of vulnerability even in times of perceived economic progress. Perhaps a further postulation would be that women migrant workers in Africa face core hardships than their counterpart women migrant workers in regions and continents that have stronger institutions and more gender inclusive policies and practices.

Within this rubric, this paper explores how Covid-19 policy responses have contributed to the multiple vulnerabilities of female migrants in Southern Africa, based on life experiences of Zimbabwean female migrant workers based in Botswana and South Africa. The paper explores the personal experiences of these women, referred to in this paper as key conversants, juxtaposing the vulnerabilities and challenges posed by migration to the existing opportunities to reveal how gender, the ‘shadow pandemic’, has doubly exposed female migrants to the virus while heightening their socioeconomic challenges. Existing mismatches between policy and practice in the Covid-19 response for migrants in general and for women migrants in particular can become the main focus of development practitioners globally in order for effective gender responsive practice against Covid-19 to be realised.

Findings of the research established that Covid-19 has negatively impacted on migration policy and practice, first by compounding gender discriminatory outcomes that pessimistically impact upon women migrant workers and girls more than they impact on male migrants and boys; and secondly by rendering existing migration policies outdated and sometimes inapplicable and result-less. Recommended actions include the need to continuously reflect on the impact of Covid-19 from a gender and intersectional perspective, as well as the need to couch more relevant migration policies in the face of the pandemic. Incorporating gender as a tool for migration policy and practice analysis is important for enhancing the incorporation of greater protection and support to more vulnerable populations in response plans, beyond just identifying the risks and inequalities.

## Context

Conceptually, heightened female migrants’ vulnerabilities during Covid-19 can be classified as a human rights violation perpetuated by policy makers and driven by human relational variables such as power, race, sex, ethnicity, nationality and religion. In other words, how migrants access their health needs during and post migration may largely depend on their social standing—their class, race, gender, education levels and ability to understand or speak a specific language. In Africa and elsewhere, deprived women and children are many a time placed in the same bracket and treated as minors owing to obtaining patriarchal relations. As such, formation of group disparities among such migrants places lowly skilled women, pregnant women, babies and adolescents who are migrating illegally in a group of deprived migrants as opposed to skilled and legal migrants. Macro-level factors such as systems of governance, labour market policies, social and economic policies, migrant-hostile political discourse, and culture may likewise militate more against the most vulnerable while providing support to the less vulnerable.

Firstly, as already noted in foregoing arguments, women oppression is a universal factor. Women are classified as less powerful, especially if they are low class women, pregnant women, nursing women and women who cannot understand the dominant language of communication. As a result this group of women is more discriminated against than men, and depending on their age and individual levels of resilience, most of these women fall victim to psychological disorders during and post migration. Studies on mental ill health among immigrants have found significant differences in mental health outcome between immigrants from high income countries and those from low-income countries, while the status of asylum seekers or refugees is also associated with mental ill health.

Available literature further proves that migration has had ‘differential filtering effects’ in both pre-covid and post covid epochs (Anderson, 2001). Prior to Covid-19, the legal or illegal forms of migration translated into regulated or unregulated livelihood pathways that resultantly defined the patterns of migrant exclusions and/or inclusions, at the same time dictating the bipolar life trajectories of migrant workers—either positively or negatively, depending on their legal standing. While regulated forms of migration on one hand had the potential to avail opportunities for social services and legal employment opportunities only to the legal migrants, unregulated migration shaped livelihood pathways that engendered greater risks and heightened vulnerabilities for the affected populations. The effects of these bipolar or filtering effects have been further exacerbated by the COVID-19 pandemic. Covid-19 has heightened the need for health security, health benefits and economic stability to migrant workers and their families on one hand, and on the other hand it has also determined which bracket of migrant workers and their beneficiaries have access to these needs. While regulated migrants have in most cases had access to health, food and job security during the pandemic, unregulated migrants have on the other hand (in most cases) had reduced or shut down access to health facilities, health services, job security, rights to employment benefits and accommodation (Suhardiman et al., 2021), owing to the fact that they do not exist in data bases for policy makers and resource allocators. As already noted, SDH theorists argue that it is not the specific health problems such as for example pregnancy complications, STIs or Covid-9 that kill the majority of irregular migrant women and girls, but rather their social positions as second class citizens that prevent them from accessing the health services that other women who are not positioned as irregular migrants are able to access (Gumà et al. 2019). Women are susceptible to various forms of stresses and mental disorders than men, owing to a number of reasons.

While the dividing line between regulated and unregulated migrants may appear too easy, the argument that layers of vulnerability amongst even regulated workers alone have also been increased by geographies of insecurity created by for example, contextual differences that favour migrant workers in developed countries against migrant workers in less developed countries sustains. As such, regulated migrant domestic workers in Lebanon and the Gulf states (Karasapan, 2020), like those in most Africa countries, have lost jobs due to pandemic restrictions while those in developed countries like the United States and the United Kingdom have ether retained their jobs or benefitted from benefits out aside for disaster risk management purposes (Karasapan, 2020). In Southern Africa for example, some migrants faced forced deportations back to their countries of origin where chances for adaptation and survival were already under siege and worse than before they migrated, owing to the effects of Covid-19 (Dube, 2019). For other migrants, the closure of borders resulted in displacement, isolation and exposure to more risks as most of them became destitute either in the host countries or at borders, without access to medical treatment, shelter and even food.

Migrant smuggling was likewise on the increase. While in the past migrant workers would be smuggled across the borders as a way of flouting the rules of regularization, during Covid-19, migrant smuggling increased both as a way of circumventing the more strict border requirements for returning residents, but also as the only way of overcoming obstacles in order to attend the ever increasing funerals of family members, for those migrants whose relatives die from Covid-19. The advent of the Covid-19 pandemic has compounded new problems like had never been anticipated before, and requires a new focus on how to grapple with reality when the world is threatened by the possibility of a total shut down, when economies stagnate, institutional systems are strained and development policies become a social nullity (Suhardiman et al., 2021). Covid-19 has indeed wrought a push back to the narrative of migration as global development; hence a gender analysis of the differentiated manner in which male and female migrants experienced these difficulties is indeed a case of academic and policy concern.

## Methodology

Using a gender lens, this paper analysed how the global health crisis of Covid-19 has intensified existing challenges for migrant women and their families, based on a qualitative inquiry that produced both primary and secondary data from which the research findings were deciphered. Secondary data was gathered from a review of available literature on migration books, journals, internet articles and research papers written before, during and after the advent of Covid-19. Primary data was generated from strategic conversations carried out with fifteen Zimbabwean women migrant workers based in South Africa and Botswana. Three of the research participants were purposively sampled by the researcher based on her knowledge of them as former connections in the women’s movement in Zimbabwe. The other twelve participants were recruited to participate in the research using the snowballing sampling method, where the researcher kept asking each woman to provide names of one or more women migrant workers known to them, and who they were willing to connect the researcher to for the purposes of this research. In the introduction session before the beginning of each strategic conversation, the researcher introduced herself, mentioning the name of the women who had provided the reference for the key conversant, and this helped in establishing trust and rapport between the researcher and the key conversant The researcher also took time to explain the motives of the research as well as explain that the findings would be anonymous, and the report non-attributed, before requesting for informed verbal consent from each key conversant.

Given the sensitivities surrounding the topic and the need to safeguard the emotions of participants, informal strategic conversations were used for data generation. The use of informal strategic conversations as opposed to a strict question and answer model helped the researcher to form a non-hierarchical set of relationships with the participants, referred to in this paper as key conversants. This was so because strategic conversations, unlike the formal structured or semi-structured interviews, allow unrestricted and free conversation between the researcher and the key conversant, and are guided by the relevant talking points as opposed to being pinned down to a strict set of pre-meditated questions. While strict conventional interviews have potential to instill fear and limitations, strategic conversations advance an understanding of otherwise unobservable aspects of research, as well as hold promise for further methodological innovation in qualitative research (Kyprianou et al., 2016), in that besides allowing the researcher to reach the domains of the key conversant, based on the free environment that will be obtaining, they also provide a methodological deviation from the semi-structured interviews, which in most cases is the norm for interviews. In line with the tenets of feminist epistemology, strategic conversations reduce power dynamics between the research facilitator and the key conversants. After all, the conversations proceed naturally in a mutual and collegial manner. Informed consent to carry out the strategic conversations was obtained from each individual key conversant before the beginning of each conversation. The need to anonymise the names and host countries of the key conversants was likewise agreed on, given the sensitivities associated with the topic. The research is therefore non-attributed. Key conversants are identified using alias numerical codes, ranging from KC1-KC15. The host countries of the key conversants are likewise anonymised, in line with the ethic to safeguard the interests of the women migrant workers.

## Discussions

Migrant workers are the spine of health care systems and thriving economies; working as doctors, nurses, scientists, researchers, entrepreneurs, care takers, domestic workers, cleaners, essential workers and much more (IOM, 2020a, b). In the face of the current pandemic challenges, migrant workers are at the front line of the pandemic response, and would be the first group to suffer the risks that the pandemic present. In many economies, power hierarchies are ordered by gender. As a result, more women form the bulk of either those professionals who are in closest contact with the sick such as nurses or the majority of general hand workers such as cleaners, health care workers and domestic workers, who likewise, are in closest contact with the sick. The increasing presence and dominating figures of women and girls among the migrant population has thus become an identified and crucial feature of international migration globally (Sharma, 2011). Nearly 50 percent or 95 million of the overall migrant population are women (UNFPA, 2006). Migration statistics further ascertain that over the last four decades, the percentage of female migrants is approaching half of the total migrant stock worldwide (Sharma, 2011). In Africa and Asia, although research proves that male international migrants out-number female international migrants, there is enough evidence to prove that between 2000 and 2020 the increase in the estimated stock of female international migrants in Africa (69 percent) was slightly higher that the increase in male international migrant which was pegged at 68 percent (https://www.migrationdataportal.org). In such a situation, ignoring the plight of women migrants and failing to couch gender sensitive policies that can effectively protect them from harm is similar to sentencing women and girls to global health genocide, thus a violation of human rights and an impediment to the achievement of the set goals for sustainable development.

Most importantly, the playing field is not level for all migrant workers in all institutions. Women and girls face intersecting forms of inequality and discrimination, whose impact has far reaching consequences on their well-being and on the well-being of their children and families. The situation is worse off in contexts where resources are strained and institutional capacity is limited. Such a reality is further amplified in contexts of fragility, conflict, and emergencies.

In Southern Africa, migration across borders is a result of economic pursuits, political instability and environmental crises (https://www.migrationdataportal.org). Out of its population of 363.2 million people, Southern Africa recorded a total of 6.4 million international migrants in mid 2020 (UNDESA 2017). While Zimbabweans migrate to different destinations of attraction globally, when it comes to Southern Africa, a few countries which have been identified as the key economic pillars in the country have been the centres of migration attraction for Zimbabwean migrant workers. For those migrating specifically for economic pursuit, South Africa, Botswana, Zambia and Angola have been the migration magnets for unskilled, skilled, regularized and unregularised migrants from Zimbabwe (https://www.migrationdataportal.org).

In the section below, some of the layers of vulnerabilities encountered by female migrants from Zimbabwe who reside in South Africa and Botswana, made worse by the presence of Covid-19 are discussed, in line with the findings from the secondary and primary data generated by the research. While 15 women were interviewed, findings are presented anonymously as part of the research’s safeguarding considerations. As such the key conversants were allotted identity numbers ranging in-between KC1 to KC15, which stand for key conversant 1 to key conversant 15 respectively. Since the findings are presented thematically rather than in chronology of the conversations held, the key conversants will be quoted in line with the ermeging themes.

All key conversant who participated in this research concurred that while their jobs in Botswana and South Africa have always been insecure since most of them lack the required work permits and documentation, Covid-19 has further exposed them to employment insecurity as many most of them have lost both their jobs, wages, accommodation and health insurances. To buttress this point, KC11 commented that,

I worked in a care institution. I had accommodation in the workers quarters and I was assured of my monthly wages. After the second lock-down I was summoned to the office and told that the institution was down-scaling and streamlining its workers, so I was going to be laid off. There was no chance for me to serve a notice period, and I was going to receive wages for the next three months as a lump sum to cushion me. I had to leave the institutional premises. I left and went to lodge with my distant cousin who rents a house in the city. I have two minor children who live with my aunt back home, so I sent all the money for their school fees and upkeep in advance, since I knew that I would not get any money in a long time, given the uncertainties of the pandemic. I am facing a lot of challenge now, regarding my accommodation and upkeep. I have burdened my cousin, who also cannot afford to look after me because although she is still working from home, her salary has been cut by half. I cannot find another job easily, but I have joined an online course to learn more about care work, with the support of my cousin who paid the online fees for me. I am hoping that I will get a placement but the chances are slim gen that institutions are streamlining workers.

The experiences of KC11 resonate with the experiences of many other women migrants in Southern Africa, across the regions and globally who are facing the situation of insecure jobs, loss of jobs and loss of income across formal and informal institutions globally (Dube, 2019). In most cases, where workers have to be laid off to offset financial imbalances created by a pandemic situation, it is women in menial job positions who get laid off first. The majority of female migrant workers are concentrated in the lowest rank, where they provide essential services in lowly paid positions as domestic workers, care takers for the sick and elderly, laundry workers and cleaners. This reality likewise confirms foregoing research findings that in developing economies 70 per cent of women’s employment is in the informal economy where there are few or no protections against dismissal, no paid sick leave, no health insurance and limited access to social security and related protection schemes (Boniol et al., 2019).

For KC4, the major issue after she lost her job in the hair salon where she was working as a hairdresser was how to go back to Zimbabwe.

I worked as a domestic worker. I lost my job at the onset of the pandemic, during the first lockdown. I could not get enough money to relocate to Zimbabwe after the lockdown. The little savings that I had when we closed shop in the first lockdown got used up on food and rent during the first lockdown. After the lockdown there was need for money to do the Covid-19 tests that were required for me to get clearance at the border. There was also need for me to meet transportation costs for my goods and for my journey back home, but I could not afford that. I sold all my property and got enough money to move from the city where I worked and lived for six years, hoping that if I got to a city closer to the border I would be able to cross over into my country, but my arrival coincided with another lockdown and I had to look for temporary lodgings in 2021. Since then I am still stuck here, and the money is used up. I have resorted to vending vegetables in the locations just to get enough money to survive and share rentals with other women. I have not been able to send money back home to my mother who looks after my kids in a very long time, and I know life is very difficult for them.

The Covid-19 lockdown restrictions saw many migrant domestic workers being dismissed from their jobs and becoming destitute in foreign countries as they cannot cross closed borders to return back to their countries of origin (IOM, 2020a, b). The International Organisation on Migration (IOM) estimates that there are 67 million domestic workers in the world, 80 per cent of whom are women and 11.5 million of whom are migrants (IOM, 2020a, b). The domestic workers sector has always been one of the most marginalized, least protected, least secure, most violent and least valued employment sectors before the pandemic, which became worse after the onset of Covid-19. The International Labour Organisation (ILO) further estimates that Covid-19 could cause 25 million jobs to be lost globally in the next few years if a cure is not found, and of these 25 million job losses, women migrant workers are estimated to be the hardest hit (UNDP and UN Women, 2021).

KC15 worked as a ticket sales officer for two cross border bus companies. Following the travel restrictions, she got laid off unexpectedly.

I had no savings, no health insurance and no alternative source of income. My stay was illegal so I could not even appeal for assistance from that country. I was 7 months pregnant, my husband was not working and we have another 4 year old child. 3 other children were living in Zimbabwe with my mother who is a widow. I had registered for prenatal checks at a state hospital but that same month it was gazette that all foreigners would be required to pay an exorbitant fee in order to be admitted for maternity health services. I did not know what to do under the circumstances. I traded in all the household property that we had for a paltry sum just to take me back home. I knew I would be able to get free maternity services in the state hospital back home. Just arriving back into the country without preparations was the hardest thing that I did, and my family bore the hardships. We had to squeeze into my mother’s two roomed lodgings with all the children, and my husband went to live with his mother’s family. I have finally managed to give birth to a baby boy but ends cannot meet and all my other children are not in school since last year. We live hand to mouth from the vending business that I found my mother doing and I have since joined her. Life is so complicated and when we get sick from flue and other diseases we resort to traditional remedies such as steaming and herbs.

The majority of women migrant workers who occupy low paying jobs in both the formal and informal sectors are by default excluded from social protections and related insurance schemes. This makes them lose income benefits and other socio-economic safety nets, further hindering their access to health care services, and the situation is worse for unregulated migrants. The cross border bus services thrived so much on the movement of cross border traders in-between countries during the normal times. More than 200, 000 Zimbabweans are on record for having returned home due to the economic fallout imposed by Covid-19 on their host countries (IOM, 2019).

The reallocation of resources and priorities, including resources for sexual and reproductive health services in host countries during the Covid-19 period is also another women specific challenge, yet the provision of sexual and reproductive health services, including maternal health care and gender-based violence related services, are central to health, rights and well-being of women and girls. The diversion of attention and critical resources away from these provisions may result in exacerbated maternal mortality and morbidity, increased rates of adolescent pregnancies, HIV and sexually transmitted diseases. This challenge was not peculiar to Southern Africa alone, as evidence from Latin America and the Caribbean indicates that an additional 18 million women will lose regular access to modern contraceptives, owing to the prevailing Covid-19 pandemic context (UNDP and UN Women, 2021).

While the most recent World Economic Forum gender gap report projects that it will take an approximate 136 years to close the gender gap, what it means in the face of Covid-19 is that the gains achieved so far in the area of women’s rights since Beijing in 1995 will continue to be rolled backwards. The impacts of Covid-19 have potential to roll back the brittle gains made so far in the area of female labor force participation, and are a double edged sword for female headed households where loss in income equates total loss of livelihoods and financial security (Erasmus, 2020). Prevailing estimates are that lockdown measures have by now affected almost 2.7 billion workers, making up approximately 81% of the world’s workforce (https://www.ilo.org/global/about-the-ilo/newsroom/news/WCMS_740893/lang-en/index.htm). Against this evidence, the International Monetory Fund (IMF) on the other hand highlights a significant contraction of global output in 2020, and that Covid-19 is pushing the world economy towards a global recession which will be strikingly different from past recessions (IMF, 2020).

The experiences of KC2 and KC 12 showcase how SDH including gender, ethnicity and socioeconomic status account for the multiple or intersecting inequalities among migrant workers during crisis periods. KC2 had this to say,

I lost my mother to cervical cancer during the lockdown. I brought my mother into this country specifically for health reasons. She had no one else to look after her in my absence, and also, health care services were better accessible here than at home. Before the pandemic I was in contact with many health care providers who provided my mother with medications, care services and other relevant information mostly for free. After the pandemic most of those providers were not in reach, were closed up or were too overwhelmed to attend to my mother. My salary had been cut by half and I could not afford private hospital charges. Taking her to the state hospital was a nightmare under the conditions that were prevailing; my mother was an illegal immigrant who had overstayed in the country on the basis of her health needs.

KC12 likewise had a premature birth indoors, which resulted in the death of the fetus and continuous vaginal bleeding for her because she could not access medical health on time.

I live on the outskirts of the city, and transport was difficult to hike during lockdown. I got sick in the night and had a premature baby that later passed on after two hours because I had no clue how to help myself and the baby. I bled for a very long period, even after getting medical attention.

In general, women and girls have gender specific and more complicated health needs—such as the sexual and reproductive health issues. As such unless they live in their own countries of origin or have adequate residence papers in foreign countries, they are less likely to have access to quality and adequate health services because of operating rules and regulations for access to social services. It does not matter how strong the host country is in terms of institutional and material health benefits schemes put in place to mitigate against the negative economic impacts of the pandemic because lack of valid residents documents make it virtually impossible for many migrant workers to access such schemes. The pandemic has also widened the conflict fissures between and among nations, manifesting in the manner migrants from specific countries got affected by adhoc migration policies, rules and regulations during the lockdown. Zanka and Moyo (2020). opines that South Africa took advantage of the pandemic to advance a securitized and militarised immigration policy such as the construction of a wall along the South African-Zimbabwe border, to prevent illegal immigrants and infected people from crossing over into the country. As such, xenophobic attacks resulting in loss of human lives and destruction of property and livelihoods have always been cause for concern in South Africa.

During a health crisis period such as Covid-19, difficulties caused by prevailing social norms and gender stereotypes can also aggravate the difficulties that women have to access health services. Such was the case with KC5 who could not access the vaccines in time because of restriction from her husband that was driven by gender bias,

Each time I told my husband that I wanted to get vaccinated he would threaten me with divorce. He said the vaccine was not scientifically proven and all vaccinated people were going to die after two years. We ignored the calls for vaccination until I got very sick. This is when I got hospitalized and later on vaccinated. I discovered that as a woman I do not have to leave decisions for my health to a man even if he is my husband.

Notwithstanding the fact that they are the most affected by the virus, women are often not included in national or global decision-making on the response to Covid-19, owing to gender discrimination and patriarchal standards that govern most institutions.

A survivor of gender-based violence, KC9, narrated how she sustained serious head injuries from a domestic squabble that ensued between her and her husband.

I am a professional graphic designer and I was working in a company that was established by a fellow Zimbabwean here. My husband is naturally violent, but I never used to argue with him much because we spent most of the time separated from each other when we were still working. During the lockdown he started accusing me of so many false things every day. I got tired of these accusations until I told him that he was free to move out and leave me in the flat since I was paying the bulk of the rent. He got infuriated and violently banged my head against the steel cupboard until I sustained deep wounds. The noise from our scuffle alerted the neighbours who called in the police and they found me bleeding helplessly on the floor. I got stitched up at the local hospital; fortunately my medical aid was still functioning. These days I live in fear because he is always infuriated, ad also I know that he is not afraid of the police because after violating me he only spent two days in the cells and got released.

KC6 encountered her ordeal of gender based violence at the border, when she attempted to travel back home for her brother’s funeral during lockdown.

I had no choice but to turn back to this place. I vowed that I would never attempt border jumping again, until I am able to go back home again. When I got to the border post I could not produce money to bribe the officers, so one of them asked me to exchange sex for favours to cross over. I refused and turned back. It was a painful experience.

Quarantine conditions force women to be restricted in the same spaces with their abusers or potential abusers. Poverty is an ascertained determinant for gender based violence (GBV) in the domestic sphere, and situations of desperation wrought by loss of income and jobs are a catalyst for GBV. The ensuing section proffers insights on how the current situation of migrant populations can be enhanced.

## Conclusion

This analysis, which sought to explore to what extent Covid-19 policy responses have exacerbated the already existing multiple vulnerabilities of female migrants in Southern Africa, has successfully highlighted the difficulties that were faced by migrant populations, and especially women to access good health services and social protection services before the Covid-19 pandemic. The analysis further highlighted how the formally existing challenges for migrant populations now intersect with the pandemic wrought challenges to further positions migrant populations in different levels of vulnerabilities, and the situation is worse for women and girls. By proffering a number of recommendations for bettering the situation of migrant workers, the analysis highlighted the huge fact that responding to the pandemic is not just about rectifying long-standing inequalities, but also about building a resilient world in the interest of everyone, targeting women and girls as the key population to be positioned at the centre of recovery.

## Policy recommendations

As a requirement of human rights law, which is an important basis for protection of migrants, addressing health vulnerabilities can only be effective and complete when there is compliant engagement with gender sensitive international, regional and national laws and policies. Universal human rights instruments articulate core principles and obligations that States have to protect the human rights of all citizens within their territory. These laws give migrants equal status before the law, regardless of their migration status, further putting them at par with all other citizens in the area of individual peace and security. For example, Article 12 of the United Nations (UN) International Covenant on Economic, Social and Cultural Rights (IESCR), emphasises that the right to health extends to migrant populations, including asylum-seekers and illegal immigrants. In support of this, General Comment 14 on the ‘The Right to the Highest Attainable Standard of Health’ (the Right to Health) moves beyond just addressing issues of access to health services toward inclusion of a human security based health vulnerability awareness that recognises the underlying SDH such as social status, gender, living conditions, occupational health, impoverishment and discrimination. The Annex of the Health, Health Systems and Global Health discussion paper of the 2nd Global Consultation on Migrant Health further espouses other additional key instruments of human rights and international, as well as key regional policies. Needless to mention that the laws provide for the protection of all persons, but also quick to note that in a gender imbalanced world which thrives on mainstream institutions, simply referring to all persons without making specific provisions for women and girls makes women’s rights issues fall through the cracks. Perhaps the major task is how to ensure that such laws are aligned to gender specific needs of both women and male migrants as heterogeneous subjectivities, in addition to ensuring that the laws are ratified and domesticated. Simply ratifying laws without domesticating them renders those laws only persuasive and unfit to bind policy makers to implement, further making it practically difficult to hold leaders accountable for violation of human rights as a correctional measure and incentive to ensuring progress.

Practically on the social front, coherent public policy responses are required, involving the health, education, social, welfare, and finance sectors, and this can only be successful when matched with a political willingness to support this agenda. Working across sectors, policy makers have an obligation to ensure that the health aspects of migration are considered in the context of broader government policy and in engaging and collaborating with other sectors to find joint solutions that benefit the health of migrants. Leaving this task to the health sector alone may limit development creates gaps that only other sectors such as the private and business partnerships sector can bridge, for example by ensuring that adequate resources ate pooled together for a common good. This can be strengthened by widening the research base for migrant health information. Existing models for migrant health migrant policies have proved to be exclusionary in many cases because they have been based on experiences from a few countries on the globe. Bearing in mind that each context has different health dynamics and needs, there is need to increase knowledge sources and methods of inquiry to produce theories and evidence that can be easily generalized across the whole global sphere. Development of the Migrant Integration Policy Index (MIPEX) has of late enabled an effective evaluation of policies to promote the integration of migrants through a multi-pronged method of collecting information on circumspectly defined and standardised indicators across 40 countries on health care needs, entitlements, access, and responsiveness for migrants. Expanding the indicator basket for the MIPEX by increasing the number of countries of focus for the study could help make the findings more generalisable to a wider global migrant population. In the face of the pandemic, relevant research data that tackles the gender specific issues in the management and response to the pandemic must also be considered as key.

The Secretary-General’s Call to Action on Human Rights, if adhered to by all countries, can have many positive ramifications for the welfare of migrant populations in the face of Covid-19. While most governments were caught unawares and without economic safety nets by the pandemic, there is need to quickly design gender sensitive economic packages for migrant populations, and especially for women who bear the brunt of care work and food provision in the domestic spheres. Long term social security packages that can supplement the incomes of migrant workers during crises can help provide for the needs of migrant populations as well as for the needs of their extended families back in their countries of origin. It will be important to apply an intentional gender lens to the design of fiscal stimulus packages and social assistance programmes to achieve greater equality, opportunities, and social protection (UN 2020). A standardised norm for fiscal stimulus packages and social assistance programmes can go a long way in ensuring equal levels of safety and security for migrant workers in various spaces across the globe, thereby reducing the asymmetries of power compounded by existing SDH barriers, further reducing vertical and horizontal inequalities among migrant workers globally.

## Data Availability

All information provided in this paper is original to the author, and all information from other sources has been referenced, with proper citations provided accordingly.

## Abbreviations

GBV: Gender Based Violence
GDP: Gross Domestic Product
HIV: Human Immuno-Deficiency Virus
IESCR: International Covenant on Economic, Social and Cultural Rights
ILO: International Labour Organisation
IOM: International Organisation on Migration
IMF: International Monetory Fund
MIPEX: Migrant Integration Policy Index
UN: United Nations

## Footnotes

1 Vulnerability is defined as increased risk for poor physical, psychological, social health outcomes and inadequate health care See Grover (2013).
